# Supplementary material for: Greater wax moth control in apiaries can be improved by combining Bacillus thuringiensis and entrapments
Source: Nat Commun. 2023 Nov 4;14:7073. doi: 10.1038/s41467-023-42946-4 (PMC10625538; doi:10.1038/s41467-023-42946-4)
Supplement: Supplementary file 1 — Supplementary information [file 41467_2023_42946_MOESM1_ESM.pdf]

Supplementary information

Greater wax moth control in apiaries can be improved by combining *Bacillus thuringiensis* and entrapments

Bo Han[1,7], Li Zhang[1,7], Lili Geng[2,7], Huiru Jia[2,7], Jian Wang[2], Li Ke[1], Airui Li[1], Jing Gao[1], Tong Wu[1], Ying Lu[1], Feng Liu[3], Huailei Song[4], Xiaoping Wei[5], Shilong Ma[6], Hongping Zhan[5], Yanyan Wu[1], Yongjun Liu[1], Qiang Wang[1], Qingyun Diao[1], Jie Zhang[2], Pingli Dai[1] ✉

[1]State Key Laboratory of Resource Insects, Institute of Apicultural Research, Chinese Academy of Agricultural Sciences, Beijing 100193, China. [2]State Key Laboratory for Biology of Plant Diseases and Insect Pests, Institute of Plant Protection, Chinese Academy of Agricultural Sciences, Beijing 100193, China. [3]Jiangxi Institute of Apicultural Research, Nanchang 330201, China. [4]Shanxi Agricultural University, Taiyuan 030006, China. [5]Modern Agricultural Development Institute, Guizhou Academy of Agricultural Sciences, Guiyang 550006, China. [6]Enshi Academy of Agricultural Sciences, Enshi 445002, China. [7]These authors contributed equally: Bo Han, Li Zhang, Lili Geng, Huiru Jia. ✉ e-mail: daipingli@caas.cn.

Supplementary Figures 1-22

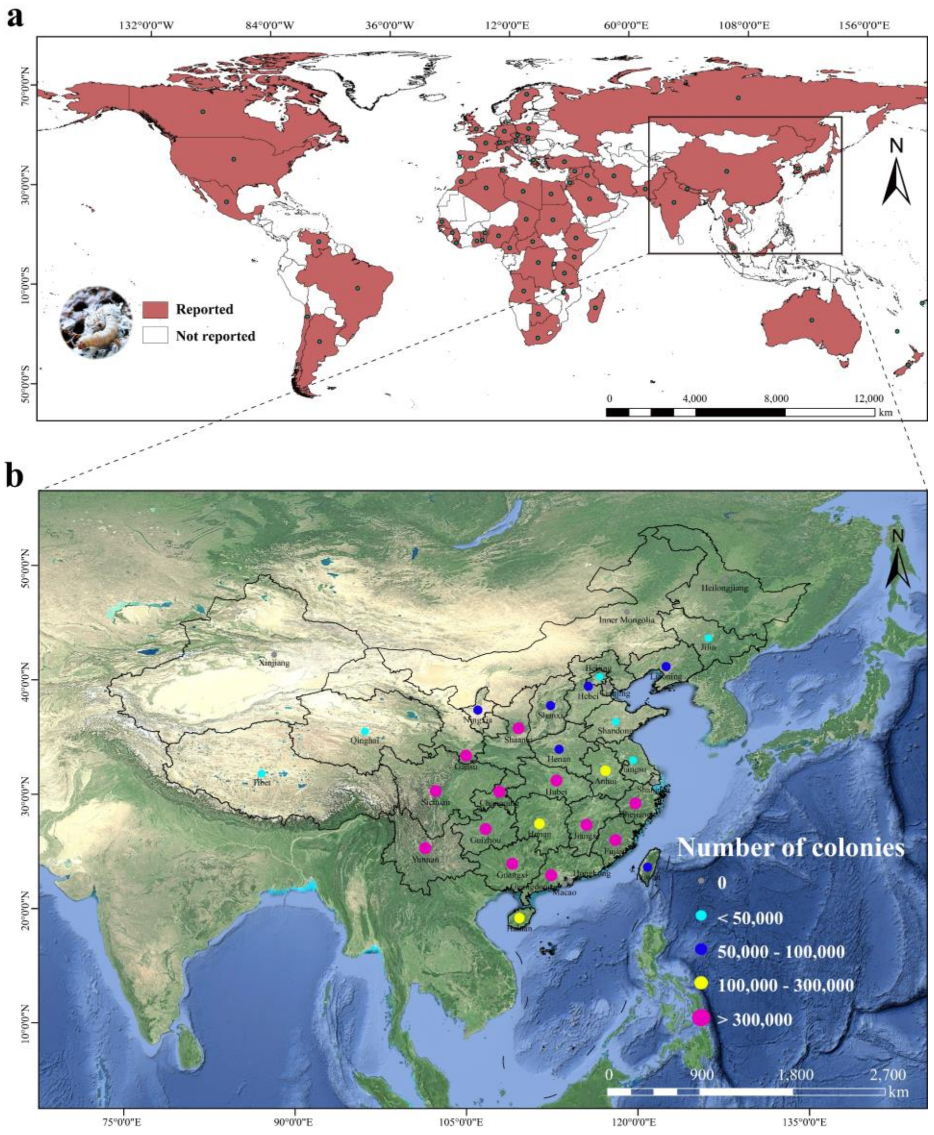

Supplementary Figure 1. Life stages and current damage status investigation of *Galleria mellonella*. a Representative images of the GWM at each developmental stage. **b** Worldwide distribution of the GWM. **c** Distribution of *A. cerana cerana* rearing colonies in China. Different colors or sizes indicated different population sizes. The original world map in the background was from Google Map (https://maps.google.com/). The original visible Chinese satellite imagery was from Google Earth, CNES/Airbus, DigitalGlobe, and Landsat/Copernicus. Map data ©2022 Google.

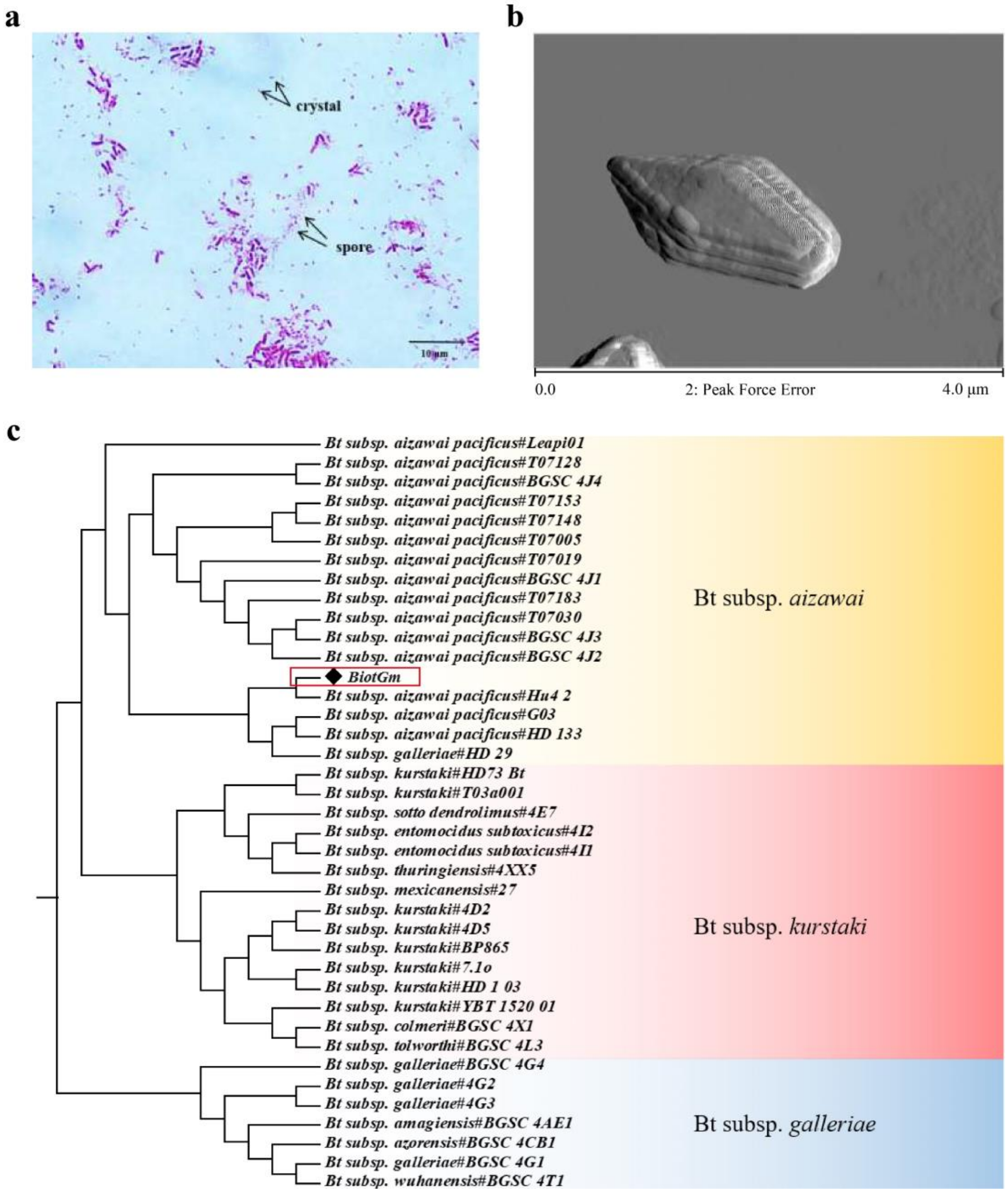

Supplementary Figure 2. Images of crystals and CVTree phylogenetic analysis for BiotGm.

Images of crystals obtained using optical microscopy (**a**) and atomic force microscopy (**b**). **c** CVTree phylogenetic.Scale bar: **a** 10 μm; **b** 4 μm.

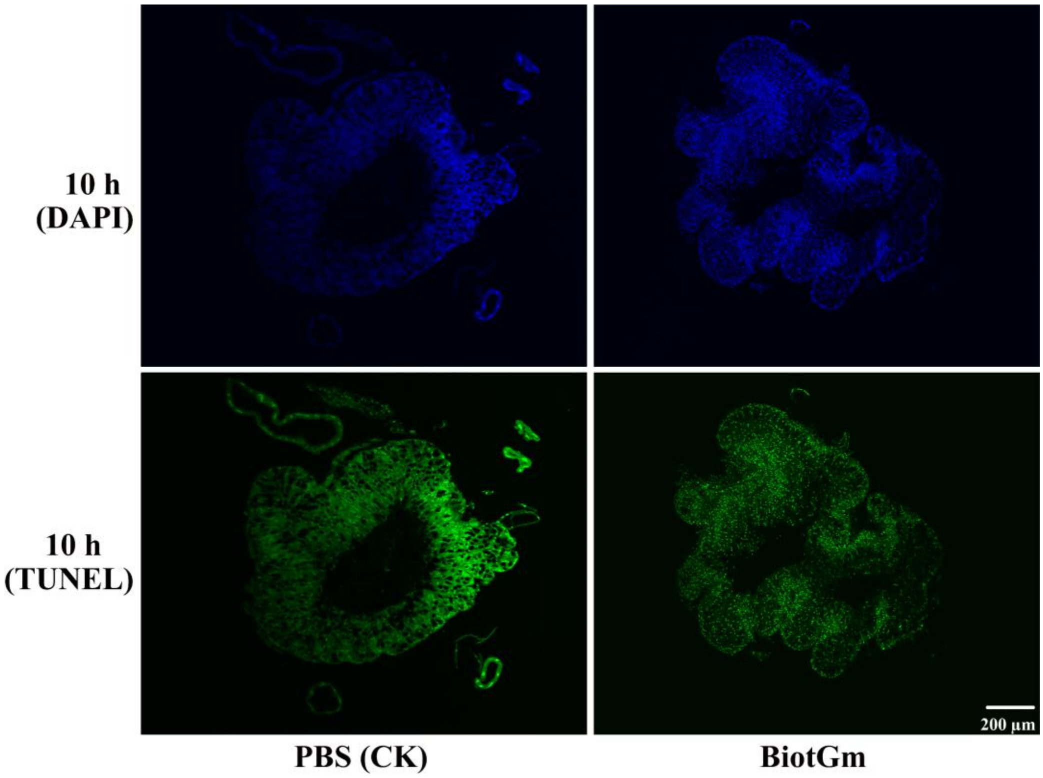

Supplementary Figure 3. Complete DAPI (blue) and TUNEL (green) stained midgut structures of GWM larvae treated with PBS (CK) and BiotGm for 10 h. Scar bar: 200 μm.

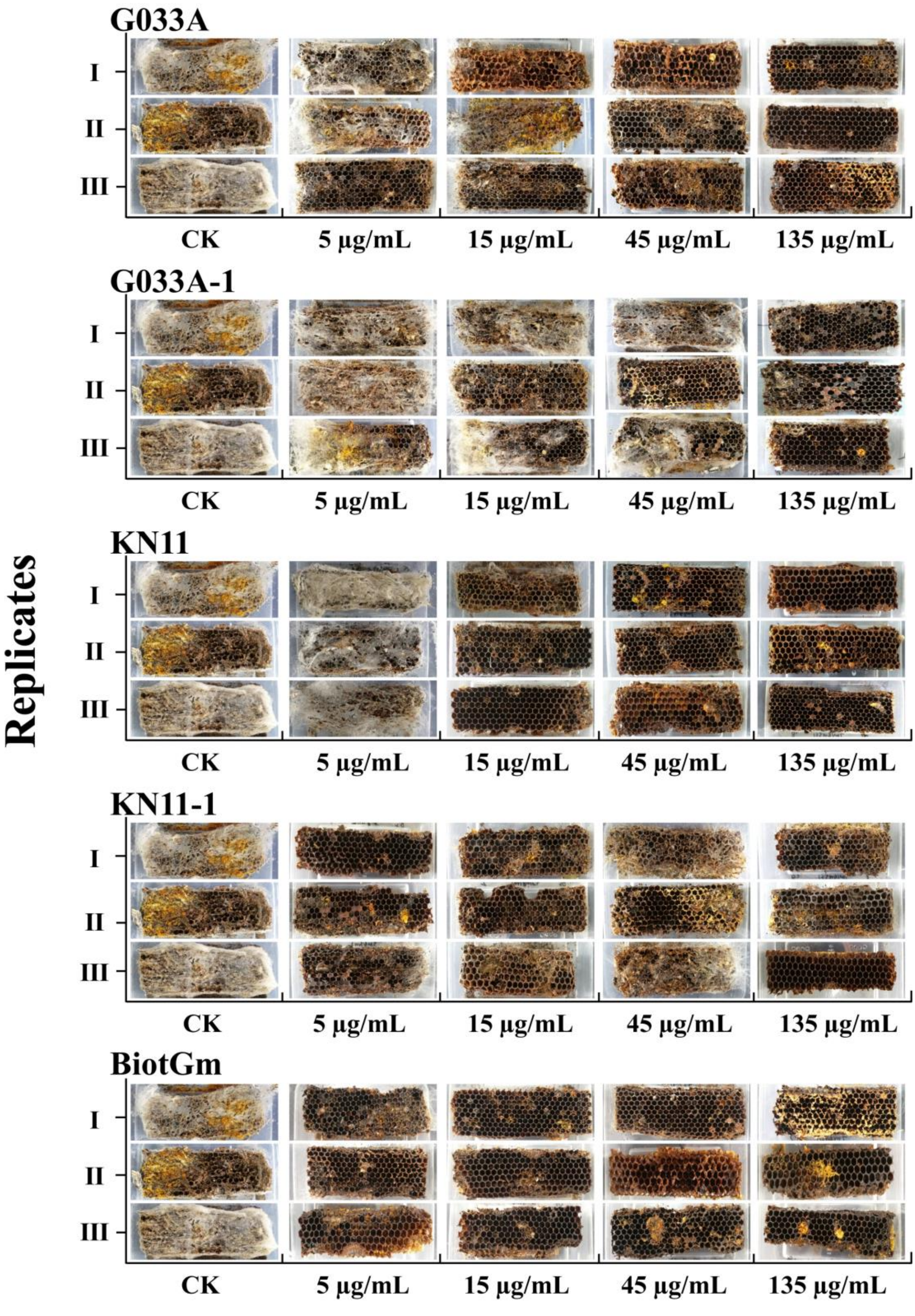

Supplementary Figure 4. The actual destruction situation of combs infested by 2nd instar GWMl larvae for 4 weeks and sprayed with 5, 15, 45, and 135 µg/mL Bt.

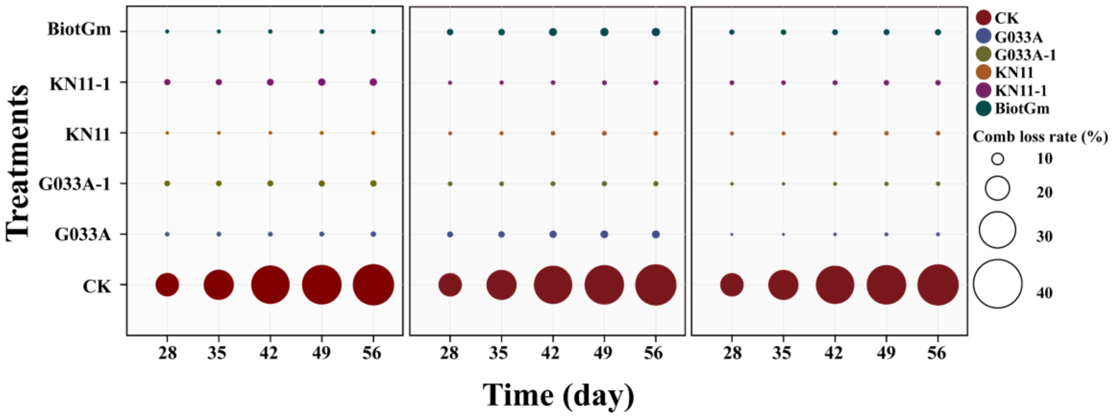

Supplementary Figure 5. The loss rates of combs sprayed with 1000, 2000 and 4000 μg/mL Bt solution and invaded for 4, 5, 6, 7 and 8 weeks by 3rd instar GWM larvae. Bubble area were presented as mean of comb loss rate from three independent biological replicates (each including 1 12 × 4 × 2 cm comb and 10 larvae). Comb loss rate data was replaced by arcsine square-root (sqrt) transformation before performing a two-sided unpaired student's t-test to analyze the differences between groups. Source data are provided as a Source Data file.

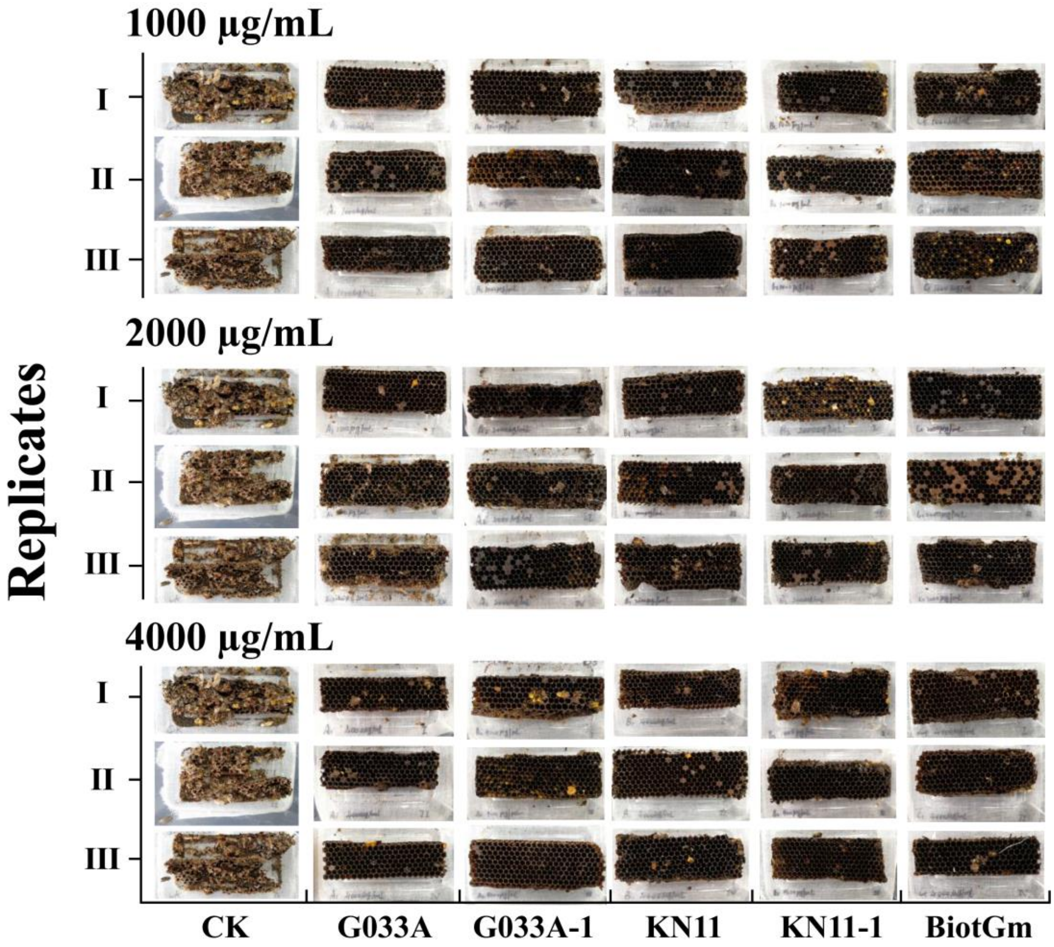

Supplementary Figure 6. The actual destruction situation of combs infested by 3rd instar GWM larvae for 8 weeks and sprayed with 1000, 2000 and 4000 µg/mL Bt.

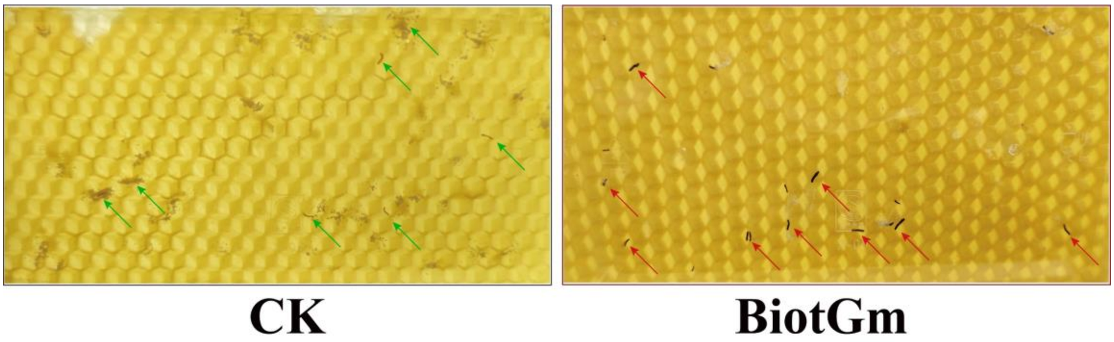

Supplementary Figure 7. The actual insecticidal activity against GWM larvae of comb foundations pressed with 4000 µg/g Bt. Red arrows show dead larvae, and green arrows show surviving larvae.

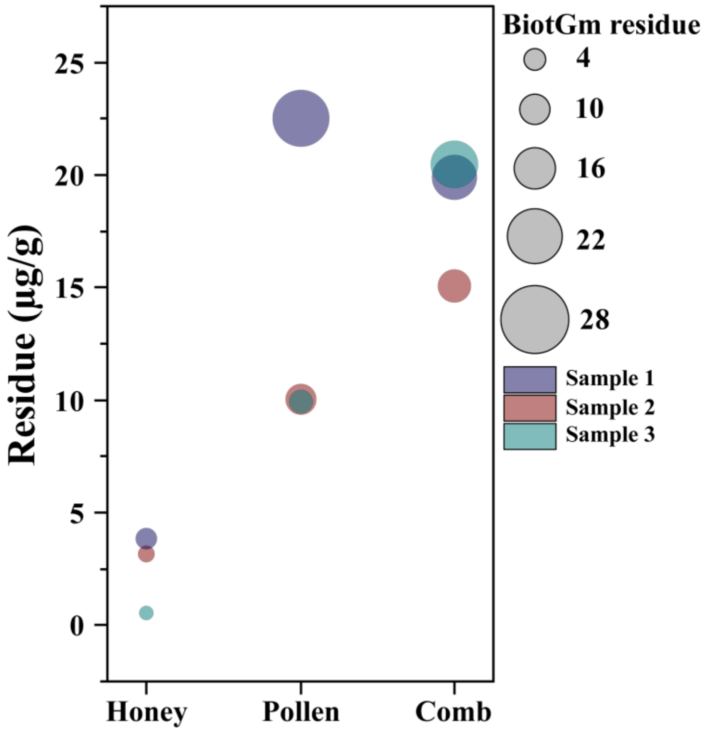

Supplementary Figure 8. Maximum BiotGm residue values in honey, pollen and comb (n = 9). Bubble area was presented as residue values of each sample. Each sample was set with three comb repeats. Source data are provided as a Source Data file.

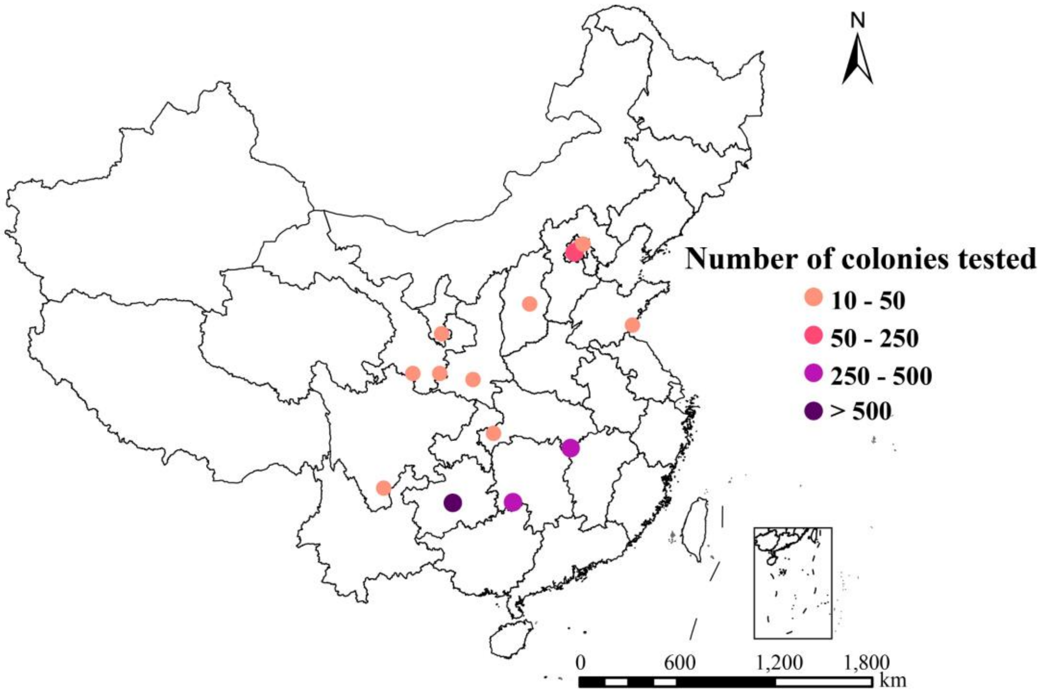

Supplementary Figure 9. Distribution of 2,950 colonies of *A. cerana cerana* from the 14 test sites in China for field trails. Different colors indicate number of colonies for testing in different test sites. The original base map of China was acquired from the Resource and Environmental Science and Data Center of the Chinese Academy of Sciences (https://www.resdc.cn/Default.aspx). Source data are provided as a Source Data file.

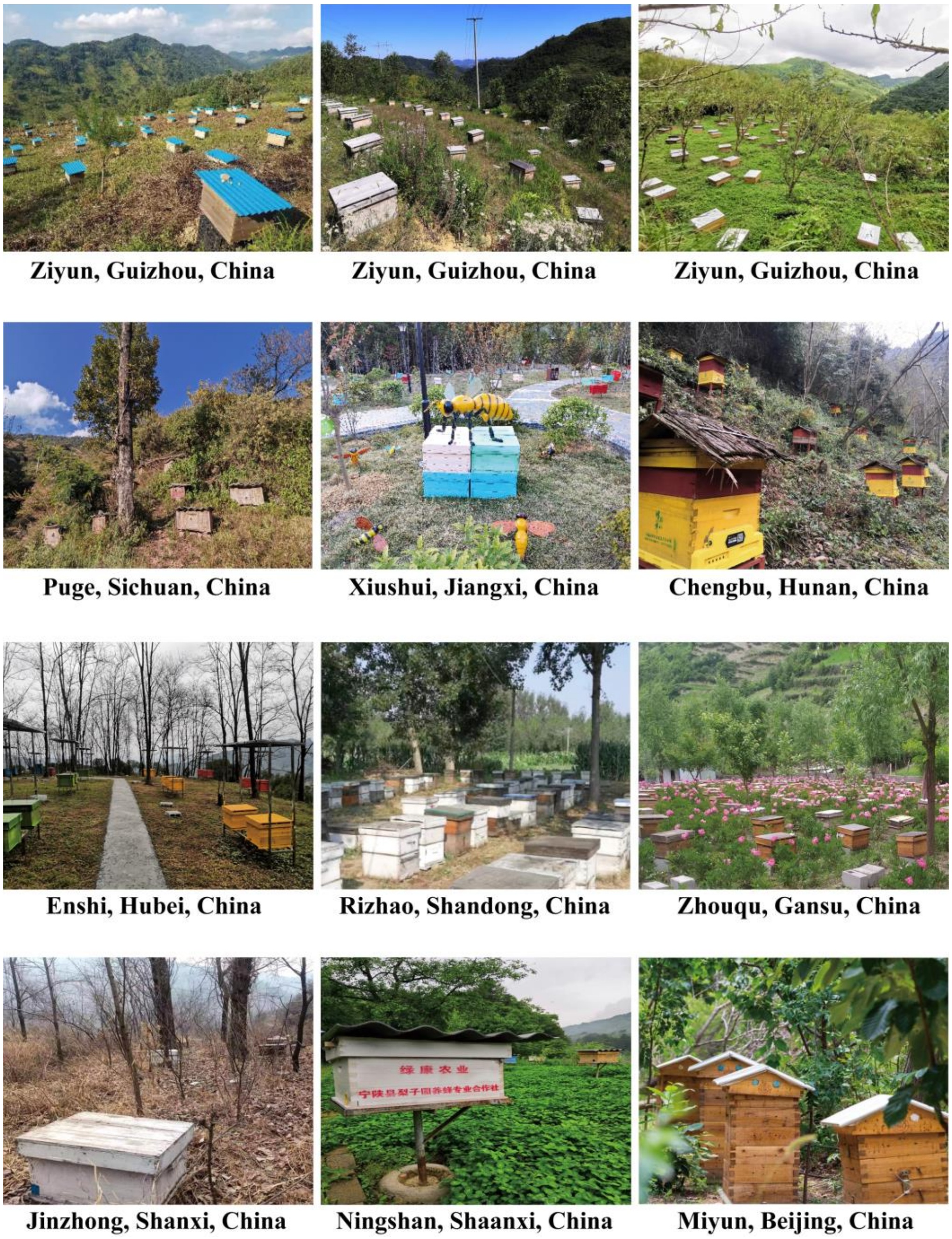

Supplementary Figure 10. The *A. cerana cerana* apiary from 14 different test sites using biocontrol entrapment to control the GWM.

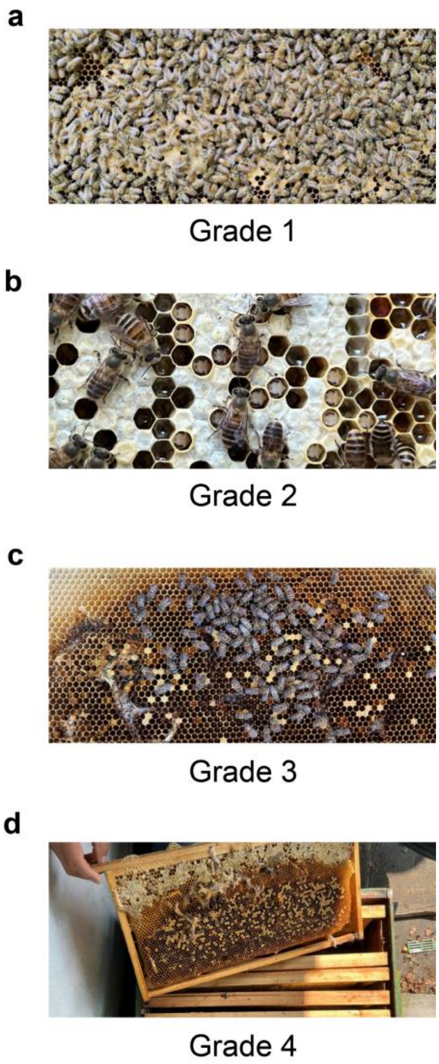

Supplementary Figure 11. Classification standard of the damage severity of GWM on colonies. The four severity levels of damage from GWM on colony including: (**a**) Grade 1, no GWM damage was found in colonies; (**b**) Grade 2, severe symptoms of bald brood (GWM larvae tunnel under wax cell cappings, causing worker bees to remove the damaged cappings) on the comb caused by GWM were identified, but the lack of observable traces indicated that GWM fed on comb and honeybee products; (**c**) Grade 3, traces (GWM larvae feed on combs, cast larval skins, pollen, and some honey) of GWM feeding on the comb and honey bee products were observed, although they did not result in colony absconding; and (**d**) Grade 4, the colony suffered severe damage from GWM and bees fled from the hive.

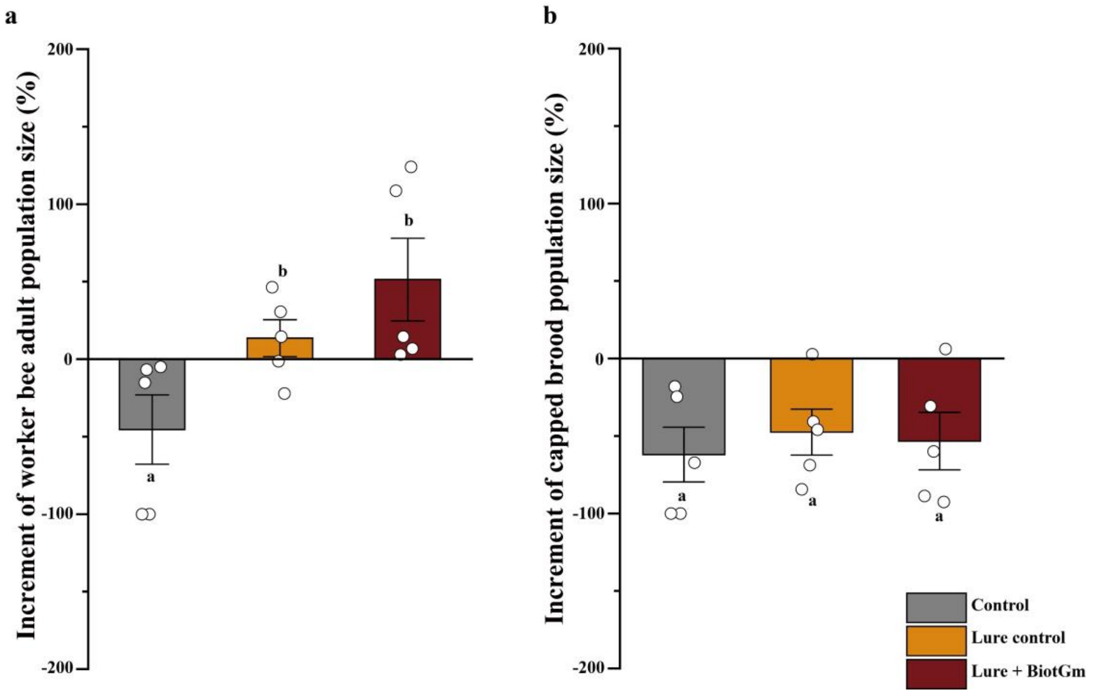

Supplementary Figure 12. Population size increment of colonies before and after the trial in different test groups from test sites of Nanchang, Jiangxi, China. **a** The population size increment for worker bee adult. **b** The population saize increment for capped brood. Data are presented as the mean ± SE of 5 independent colony replicates (**a, b**). A two-sided Mann–Whitney U test was used to analyse the significant differences between different groups of colony population increments (P > 0.05) (**a, b**). Same letters above bars indicate no significant differences between groups (P > 0.05). Source data are provided as a Source Data file.

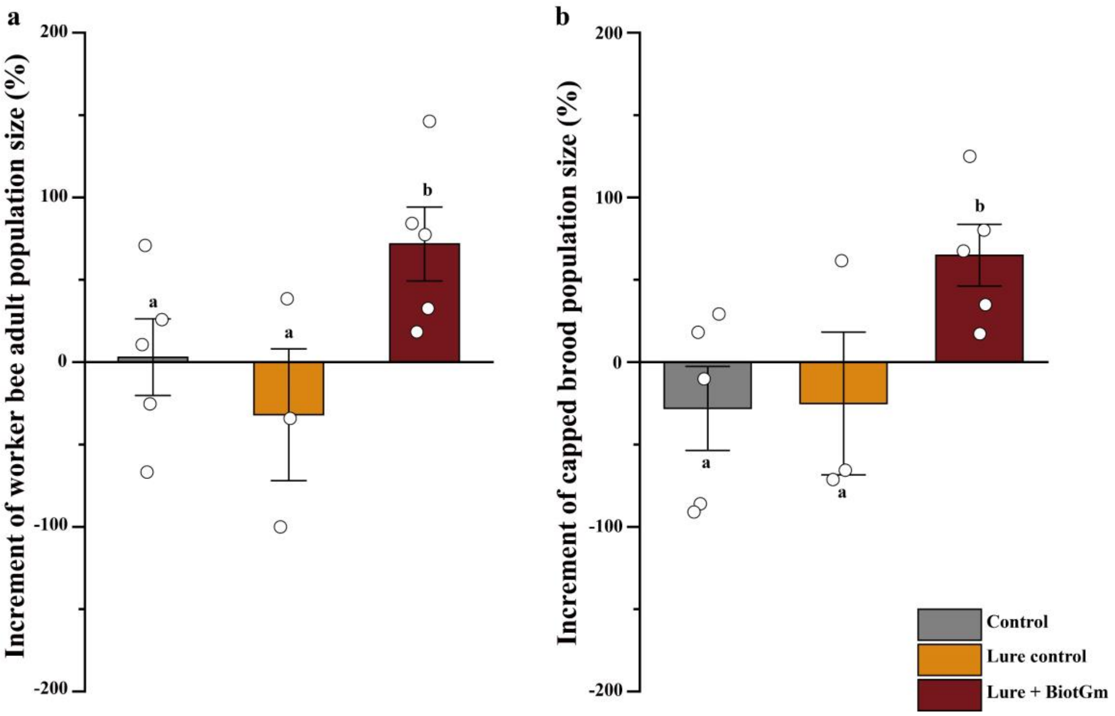

Supplementary Figure 13. Population size increment of colonies before and after the trial in different test groups from test sites of Taiyuan, Shanxi, China. a The population size increment for worker bee adult. **b** The population size increment for capped brood. Data are presented as the mean ± SE of 5 independent colony replicates (**a, b**). A two-sided Mann–Whitney U test was used to analyse the significant differences between different groups of colony population increments ($P >$ 0.05) (**a, b**). Same letters above bars indicate no significant differences between groups ($P > 0.05$). Source data are provided as a Source Data file.

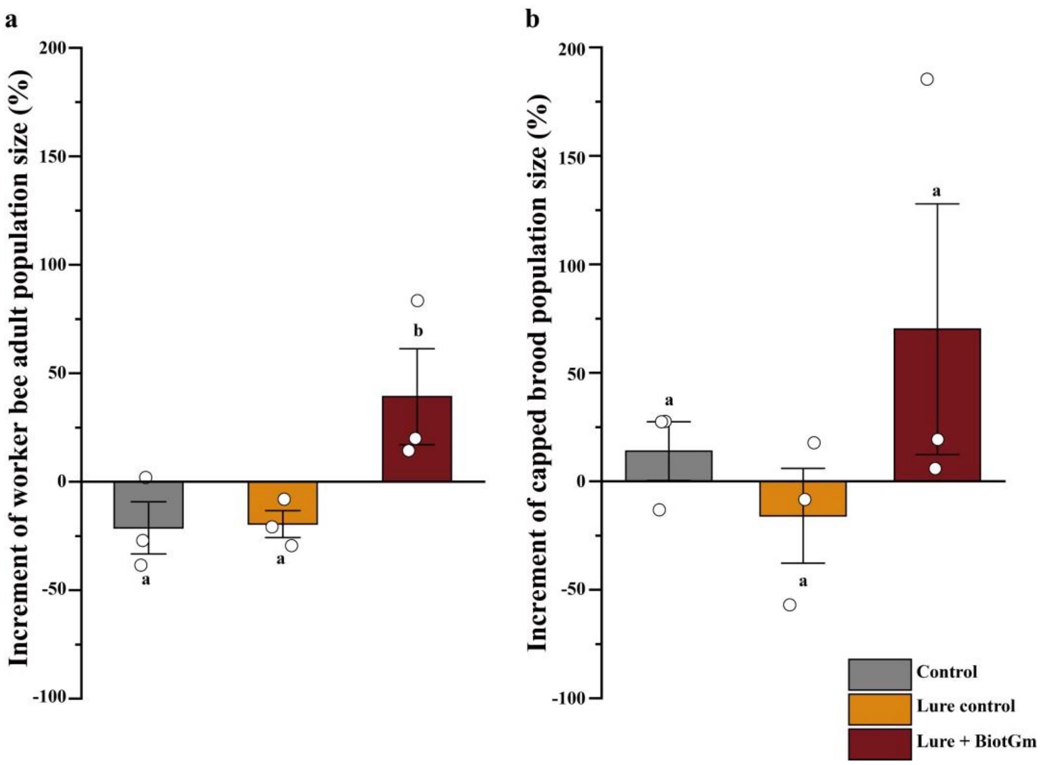

Supplementary Figure 14. Population size increment of colonies before and after the trial in different test groups from test sites of Shijingshan, Beijing, China. a The population size increment for worker bee adult. **b** The population size increment for capped brood. Data are presented as the mean ± SE of 3 independent colony replicates (**a, b**). A two-sided Mann–Whitney U test was used to analyse the significant differences between different groups of colony population increments ($P > 0.05$) (**a, b**). Same letters above bars indicate no significant differences between groups ($P > 0.05$). Source data are provided as a Source Data file.

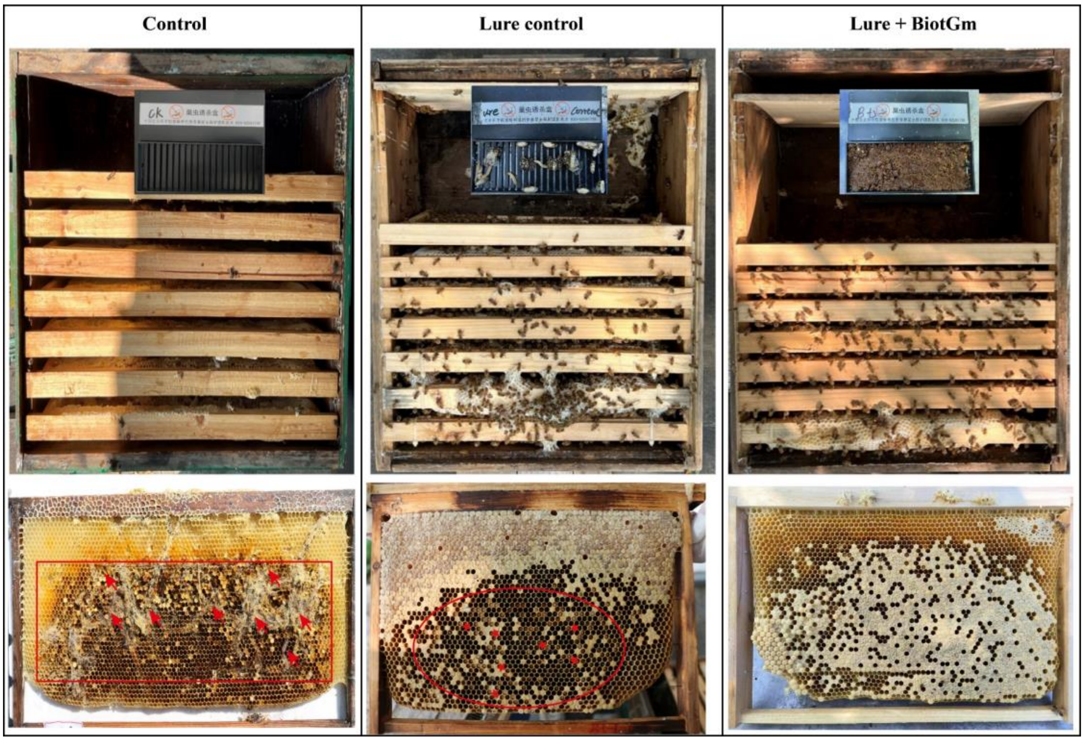

Supplementary Figure 15. Representative images of the attractive effect and control efficacy of entrapment in field conditions. The red box showed the area where the combs have been destroyed by the GWM. The The red arrow indicated the silk-lined tunnels produced by the GWM after destroying the comb. The Red ellipses represented the blad brood symptoms caused by GWM damage, and the blad broods are highlighted using red pentagrams.

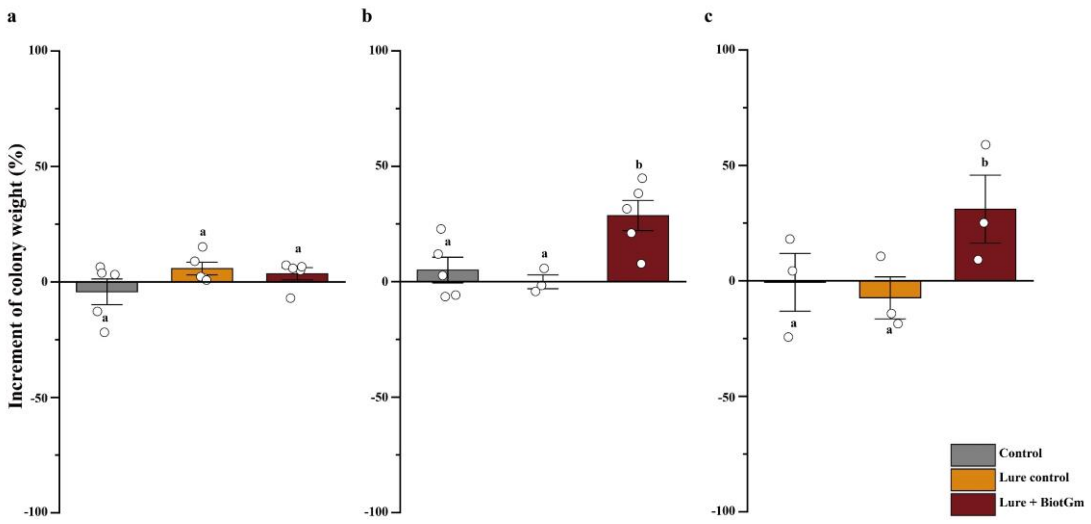

Supplementary Figure 16. Weight changes of colonies before and after the trial in different test groups from three field sites. Nanchang, Jiangxi, China (**a**); Taiyuan, Shanxi, China (**b**); Shijingshan, Beijing, China (**c**). Data are presented as the mean ± SE of 3~5 independent colony replicates for each field test site (**a-c**). A two-sided Mann–Whitney U test was used to analyse the significant differences between different groups of colony population increments ($P > 0.05$) (**a-c**). Same letters above bars indicate no significant differences between groups ($P > 0.05$). Source data are provided as a Source Data file.

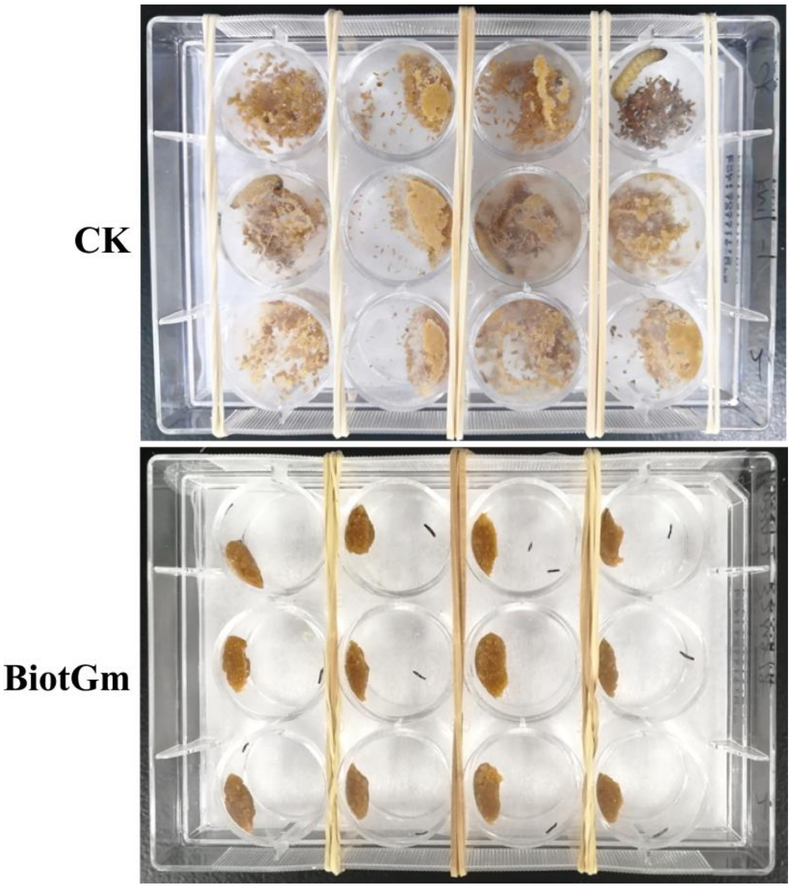

Supplementary Figure 17. The toxicity effect of BiotGm on the GWM larvae.

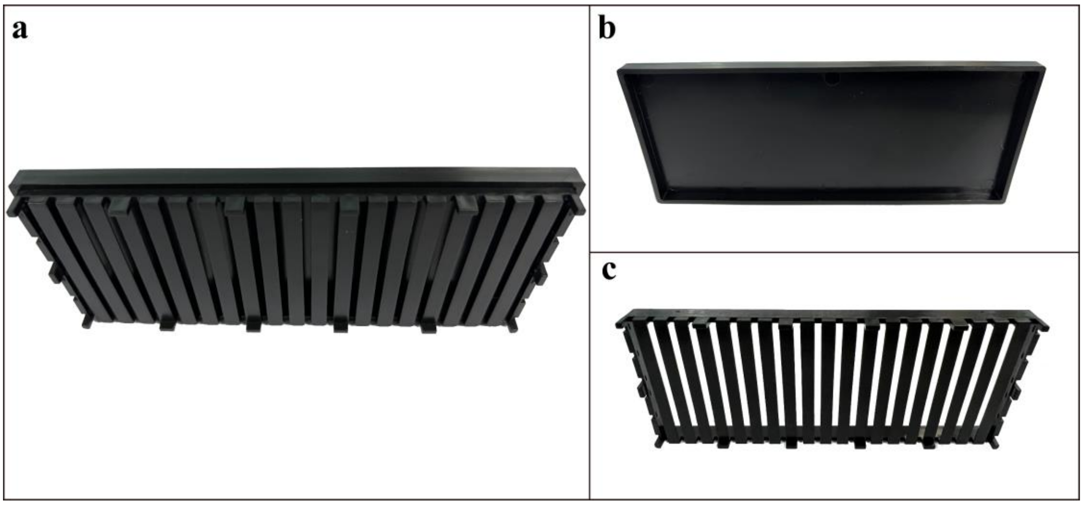

Supplementary Figure 18. Trapping device. **a** Whole image. **b** Cover image. **c** Gridded section image.

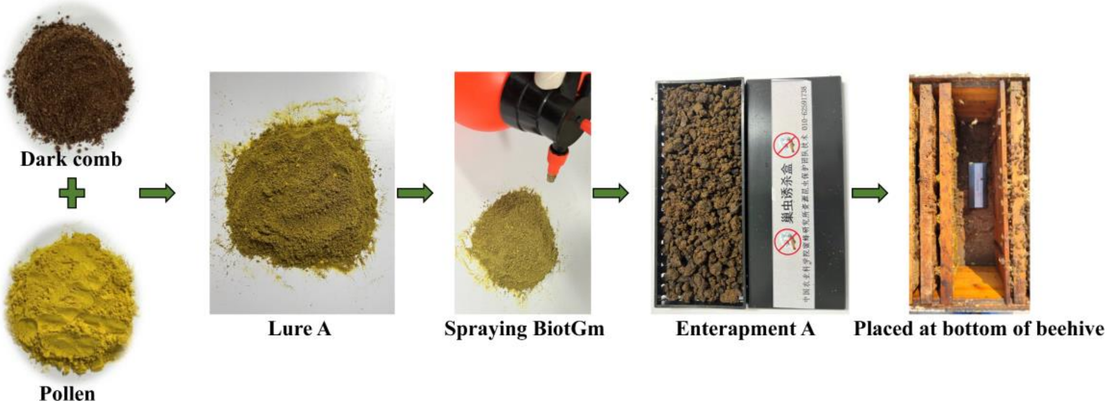

Supplementary Figure 19. The materials and fabrication process for the preparation of biocontrol entrapment.

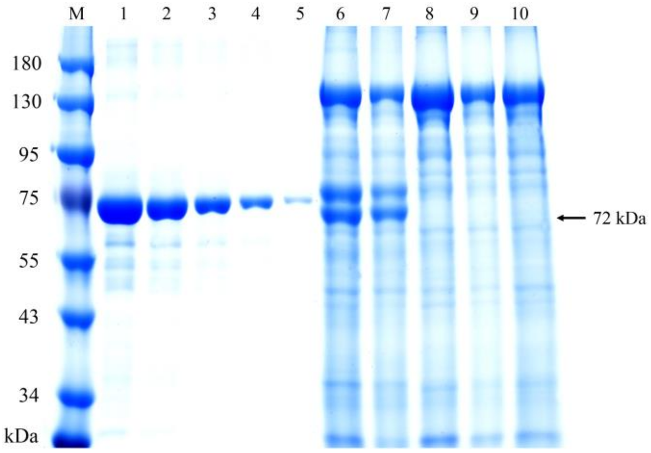

Supplementary Figure 20. SDS-PAGE analysis of total crystal proteins from five Bt products. M: Marker; 1-5: BSA concentrations were: 0.4, 0.2, 0.1, 0.05, 0.025 μg/μL; 6: G033A; 7: G033A-1; 8: KN11; 9: KN11-1; 10: BiotGm.

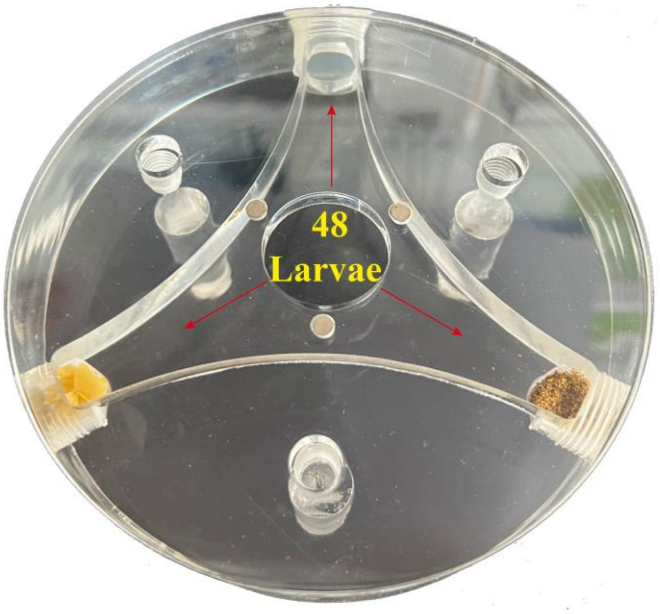

Supplementary Figure 21. The image of modified Y-type olfactometer.

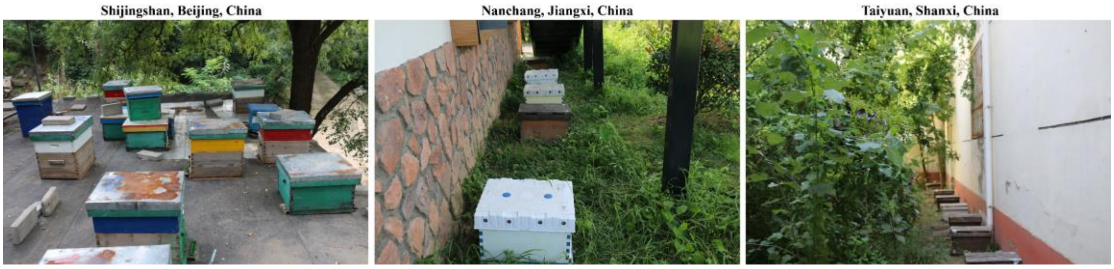

Supplementary Figure 22. Photographs of three A. cerana cerana apiary field sites. These sites were utilised to test the field effectiveness of biocontrol trapping against GWM.

Supplementary Tables 1-12

Supplementary Table 1. The sequence features of *B. thuringiensis* strain BiotGm genome

Name	GenBank accession number	Bases	GC (%)	CDS	Gene average length (bp)
Chr1	CP130743	5,718,068	35.28	6017	768
Plasmid1	CP130744	410,916	32.71	370	790
Plasmid2	CP130745	295,914	33.05	283	738
Plasmid3	CP130746	85,817	30.80	111	654
Plasmid4	CP130747	72,113	32.35	88	639

Supplementary Table 2. Annotation results of insecticidal protein of *B. thuringiensis* strain BiotGm

Protein Name	Length (bp)	Location	Annotation (GenBank No)	Amino acid similarity (%)
Cry1Aa1	3393	Plasmid2_00247	Cry1Aa1 (AAA22353.1)	99.99
Cry1Ca7	3570	Plasmid1_00270	Cry1Ca7 (AAG50438.1)	100.00
Cry1Da1	3498	Plasmid1_00266	Cry1Da1 (CAA38099.1)	100.00
Cry1Ia10	2160	Plasmid2_00248	Cry1Ia10 (AAP86782.1)	100.00
Cry2Ab1	1902	Plasmid2_00239	Cry2Ab1 (AAA22342.1)	100.00
Cry9Ea1	3453	Plasmid2_00254	Cry9Ea1 (BAA34908.1)	100.00
Vip3Aa11	2370	Plasmid2_00236	Vip3Aa11 (AAR36859.1)	100.00

Supplementary Table 3. Data analysis on survival of 2nd instar GWM larvae exposed to the comb foundation containing Bt. All survival data were analysed using Kaplan–Meier survival analysis and the two-sided log-rank test to determine differences.

Treatment	χ^2	df	*P*
CK vs G033A	168.5629	1	<.0001*
CK vs G033A-1	167.4965	1	<.0001*
CK vs KN11	159.0000	1	<.0001*
CK vs KN11-1	179.8665	1	<.0001*
CK vs BiotGm	176.4768	1	<.0001*
G033A vs G033A-1	0.1630	1	0.6865
G033A vs KN11	49.5246	1	<.0001*
G033A vs KN11-1	10.9665	1	0.0009*
G033A vs BiotGm	31.7739	1	<.0001*
G033A -1 vs KN11	46.1613	1	<.0001*
G033A -1 vs KN11-1	12.4320	1	0.0004*
G033A -1 vs BiotGm	31.7692	1	<.0001*
KN11 vs KN11-1	20.1549	1	<.0001*
KN11 vs BiotGm	14.0612	1	0.0002*
KN11-1 vs BiotGm	4.9862	1	0.0255*

Supplementary Table 4. Acute oral toxicities of BiotGm to *Apis mellifera* larvae.

Bt	*n*[a]	R^2	LC_{50} (μg/mL)[b]	LD_{50} (μg/larva)[c]	Toxic regression equations
BiotGm	360	0.986	20598.781 (17235.683-24618.102)	617.963 (520.611-733.520)	y = 3.1285x + 0.8896

[a] number of tested honey bees per treatment.

[b] Lethal concentration, with 95% confidence intervals in parentheses, causing 50% honey bee larval mortality at 72 h.

[c] Lethal dose, with 95% confidence intervals in parentheses, causing 50% honey bee larval mortality at 72 h.

Supplementary Table 5. Data analysis on survival of *Apis mellifera* worker larvae exposed to BiotGm, CK - control, Dim - dimethoate. All survival data were analysed using Kaplan–Meier survival analysis and the two-sided log-rank test to determine differences.

Treatment	χ^2	df	*P*
BiotGm 100 μg/mL vs CK	0.0000	1	1.0000
BiotGm 100 μg/mL vs Dim 45 μg/mL	133.2131	1	<.0001*
CK vs Dim 45 μg/mL	133.2131	1	<.0001*

Supplementary Table 6. Quotient (RQ) analysis. The RQs of BiotGm to *Apis mellifera* larvae were calculated using BeeREX from the United States Environmental Protection Agency (https://www.epa.gov/sites/production/files/ 2015-11/beerexv 1.0.xlsx)[a]. Maximum residues in pollen/bee bread or in nectar are derived from our result. Larval NOAEC values are derived from our data.

Bt	Maximum residue in pollen/bee bread (mg a.i./kg)[b]	Maximum residue in nectar (mg a.i./kg)	Larval LD_{50} (μg a.i./ larva)	NOAEC[c] (μg/mL)	Cumulative consumption of diet D3 – D6 (mL)	Larval NOAEL[d] (μg a.i./larva)	RQs (acute dietary)	RQs (chronic dietary)
BiotGm	22.524	3.857	617.963	100	0.14	14	0.0004	0.0583

[a] The BeeREX model was modified in order to calculate the RQ based on cumulative dose. The RQs were calculated based on the cumulative amounts of nectar and pollen that are consumed during the worker's larval phase of development according to BeeREX.

[b] a.i. = active ingredient.

[c] No observed adverse effect concentration.

[d] No observed adverse effect dose.

Supplementary Table 7. Data analysis on survival of *Apis mellifera* worker adults exposed to BiotGm, CK - control, Dim - dimethoate. All survival data were analysed using Kaplan–Meier survival analysis and the two-sided log-rank test to determine differences.

Treatment	χ^2	df	*P*
BiotGm 100 μg/mL vs CK	0.2678	1	0.6048
BiotGm 100 μg/mL vs Dim 1 mg/L	244.5654	1	<.0001*
BiotGm 100 μg/mL vs Dim 45 mg/L	236.7565	1	<.0001*
CK vs Dim 1 mg/L	221.6064	1	<.0001*
CK vs Dim 45 mg/L	231.4606	1	<.0001*
Dim 1 mg/L vs Dim 45 mg/L	231.316	1	<.0001*

Supplementary Table 8. Data analysis on survival of *Apis cerana cerana* worker adults exposed to BiotGm, CK - control, Dim - dimethoate. All survival data were analysed using Kaplan–Meier survival analysis and the two-sided log-rank test to determine differences.

Treatment	χ^2	df	*P*
BiotGm 100 μg/mL vs CK	2.8736	1	0.0900
BiotGm 100 μg/mL vs Dim 1 mg/L	197.8911	1	<.0001*
BiotGm 100 μg/mL vs Dim 45 mg/L	190.9554	1	<.0001*
CK vs Dim 1 mg/L	211.0533	1	<.0001*
CK vs Dim 45 mg/L	210.0007	1	<.0001*
Dim 1 mg/L vs Dim 45 mg/L	211.0533	1	<.0001*

Supplementary Table 9. The factual information of the 14 field test sites and efficacy feedback results using biocontrol entrapment to control the GWM.

No.	Name of beekeeper	Address	Test time	Number of colonies tested	Occurrence of greater wax moth
**1**	Xiaoli Guo	Miyun, Beijing, China (40°23'N, 116°46'E)	May to Nov 2022	10	☐Yes ☑No
**2**	Pinghong Wang	Shijingshan, Beijing, China (39°58'N, 116°8'E)	May to Nov 2022	30	☐Yes ☑No
**3**	Jinliang Liu	Miyun, Beijing, China (40°26'N, 117°1'E)	May to Nov 2022	250	☐Yes ☑No
**4**	Runsheng Yuan	Jinzhong, Shanxi, China (37°25'N, 112°34'E)	May to Nov 2022	10	☐Yes ☑No
**5**	Haizhou Wang	Rizhao, Shandong, China (35°25'N, 119°31'E)	May to Nov 2022	10	☐Yes ☑No
**6**	Jingyun Li	Longnan, Gansu, China (33°46'N, 106°4'E)	May to Nov 2022	10	☐Yes ☑No
**7**	Mao Feng	Zhouqu, Gansu, China (33°47'N, 104°15'E)	May to Nov 2022	30	☐Yes ☑No
**8**	Yong Li	Guyuan, Ningxia, China (36°0'N, 106°14'E)	May to Nov 2022	20	☐Yes ☑No
**9**	Shihong Zhou	Ningshan, Shaanxi, China (33°23'N, 108°17'E)	May to Nov 2022	20	☐Yes ☑No
**10**	Yuanhui Zhang	Puge, Sichuan, China (27°22'N, 102°32'E)	May to Nov 2022	20	☐Yes ☑No
**11**	Shilong Ma	Enshi, Hubei, China (30°19'N, 109°28'E)	May to Nov 2022	30	☐Yes ☑No
**12**	Fenghua Leng	Xiushui, Jiangxi, China (29°3'N, 114°9'E)	May to Nov 2022	10	☐Yes ☑No
**13**	Li Chen	Ziyun, Guizhou, China (26°30'N, 106°39'E)	May to Nov 2022	2000	☐Yes ☑No
**14**	Caitai Duan	Chengbu, Hunan, China (26°23'N, 110°19'E)	May to Nov 2022	500	☐Yes ☑No

Supplementary Table 10. Monitoring of the occurrence of *Galleria mellonella* in the main breeding areas of *A. cerana cerana* in China in recent years

No.	Beekeepers	Address	Survey year	Occurrence of greater wax moth
**1**	Xiaoli Guo	Miyun, Beijing (40°23'N, 116°46'E)	2020, 2021	☑Yes ☐No
**2**	Pinghong Wang	Shijingshan, Beijing (39°58'N, 116°8'E)	2017-2021	☑Yes ☐No
**3**	Runsheng Yuan	Jinzhong, Shanxi province (37°25'N, 112°34'E)	2019-2021	☑Yes ☐No
**4**	Jingyun Li	Longnan, Gansu province (33°46'N, 106°4'E)	2019-2021	☑Yes ☐No
**5**	Yong Li	Guyuan, Ningxia province (36°0'N, 106°14'E)	2020, 2021	☑Yes ☐No
**6**	Shihong Zhou	Ningshan, Shaanxi province (33°23'N, 108°17'E)	2019-2021	☑Yes ☐No
**7**	Yuanhui Zhang	Puge, Sichuan province (27°22'N, 102°32'E)	2019-2021	☑Yes ☐No
**8**	Shilong Ma	Enshi, Hubei province (30°19'N, 109°28'E)	2019-2021	☑Yes ☐No
**9**	Fenghua Leng	Xiushui, Jiangxi province (29°3'N, 114°9'E)	2017-2021	☑Yes ☐No
**10**	Li Chen	Ziyun, Guizhou province (26°30'N, 106°39'E)	2019-2021	☑Yes ☐No
**11**	Caitai Duan	Chengbu, Hunan province (26°23'N, 110°19'E)	2019-2021	☑Yes ☐No

Supplementary Table 11. The detailed information of five Bt. -1 represents the product, a commercial formulation of raw powders processed and marketed. WP - wettable powder.

Bt	Potency (IU/mg)	Registration code	Form	Total insecticidal protein content (w/w)	
				130 kDa	72 ~ 100 kDa
G033A	-	-	Primary powder	2.39	2.00
G033A-1	32000	PD20171726	WP	1.20	1.56
KN11	-	-	Primary powder	2.89	-
KN11-1	32000	PD20084969	WP	1.39	-
BiotGm	-	-	WP	2.46	-

Supplementary Table 12. The 16S rRNA-seq results of honey, pollen and comb samples by PCR.

Samples	16S rRNA-seq
**Honey_1**	AAAAGGTTACCCCACCGACTTCGGGTGTTACAAACTCTCGTGGTGTGACG<br>GGCGGTGTGTACAAGGCCCGGGAACGTATTCACCGCGGCATGCTGATCCG<br>CGATTACTAGCGATTCCAGCTTCATGTAGGCGAGTTGCAGCCTACAATCC<br>GAACTGAGAACGGTTTTATGAGATTAGCTCCACCTCGCGGTCTTGCAGCT<br>CTTTGTACCGTCCATTGTAGCACGTGTGTAGCCCAGGTCATAAGGGGCAT<br>GATGATTTGACGTCATCCCCACCTTCCTCCGGTTTGTCACCGGCAGTCAC<br>CTTAGAGTGCCCAACTTAATGATGGCAACTAAGATCAAGGGTTGCGCTCG<br>TTGCGGGACTTAACCCAACATCTCACGACACGAGCTGACGACAACCATGC<br>ACCACCTGTCACTCTGCTCCCGAAGGAGAAGCCCTATCTCTAGGGTTTTC<br>AGAGGATGTCAAGACCTGGTAAGGTTCTTCGCGTTGCTTCGAATTAAACC<br>ACATGCTCCACCGCTTGTGCGGGCCCCCGTCAATTCCTTTGAGTTTCAGC<br>CTTGCGGCCGTACTCCCCAGGCGGAGTGCTTAATGCGTTAACTTCAGCAC<br>TAAAGGGCGGAAACCCTCTAACACTTAGCACTCATCGTTTACGGCGTGGA<br>CTACCAGGGTATCTAATCCTGTTTGCTCCCCACGCTTTCGCGCCTCAGTG<br>TCAGTTACAGACCAGAAAGTCGCCTTCGCCACTGGTGTTCCTCCATATCT<br>CTACGCATTTCACCGCTACACATGGAATTCCACTTTCCTCTTCTGCACTC<br>AAGTCTCCCAGTTTCCAATGACCCTCCACGGTTGAGCCGTGGGCTTTCAC<br>ATCAGACTTAAGAAACCACCTGCGCGCGCTTTACGCCCAATAATTCCGGA<br>TAACGCTTGCCACCTACGTATTACCGCGGCTGCTGGCACGTAGTTAGCCG<br>TGGCTTTCTGGTTAGGTACCGTCAAGGTGCCAGCTTATTCAACTAGCACT<br>TGTTCTTCCCTAACAACAGAGTTTTACGACCCGAAAGCCTTCATCACTCA<br>CGCGGCGTTGCTCCGTCAGACTTTCGTCCATTGCGGAAGATTCCCTACTG<br>CTGCCTCCCGTAGGAGTCTGGGCCGTGTCTCAGTCCCAGTGTGGCCGATC<br>ACCCTCTCAGGTCGGCTACGCATCGTTGCCTTGGTGAGCCGTTACCTCAC<br>CAACTAGCTAATGCGACGCGGGTCCATCCATAAGTGACAGCCGAAGCCGC<br>CTTTCAATTTCGAACCATGCAGTTCAAAATGTTATCCGGTATTAGCCCCG<br>GTTTCCCGGAGTTATCCCAGTCTTATGGGCAGGTTACCCACGTGTTACTC<br>ACCCGTCCGCCGCTAACTTCATAAGAGCAAGCTCTTAATCCATTCGCTCG<br>ACTGCAT
**Honey_2**	GTTACCCCACCGACTTCGGGTGTTACAAACTCTCGTGGTGTGACGGGCGG<br>TGTGTACAAGGCCCGGGAACGTATTCACCGCGGCATGCTGATCCGCGATT<br>ACTAGCGATTCCAGCTTCATGTAGGCGAGTTGCAGCCTACAATCCGAACT<br>GAGAACGGTTTTATGAGATTAGCTCCACCTCGCGGTCTTGCAGCTCTTTG<br>TACCGTCCATTGTAGCACGTGTGTAGCCCAGGTCATAAGGGGCATGATGA<br>TTTGACGTCATCCCCACCTTCCTCCGGTTTGTCACCGGCAGTCACCTTAG<br>AGTGCCCAACTTAATGATGGCAACTAAGATCAAGGGTTGCGCTCGTTGCG<br>GGACTTAACCCAACATCTCACGACACGAGCTGACGACAACCATGCACCAC<br>CTGTCACTCTGCTCCCGAAGGAGAAGCCCTATCTCTAGGGTTTTCAGAGG<br>ATGTCAAGACCTGGTAAGGTTCTTCGCGTTGCTTCGAATTAAACCACATG<br>CTCCACCGCTTGTGCGGGCCCCCGTCAATTCCTTTGAGTTTCAGCCTTGC<br>GGCCGTACTCCCCAGGCGGAGTGCTTAATGCGTTAACTTCAGCACTAAAG<br>GGCGGAAACCCTCTAACACTTAGCACTCATCGTTTACGGCGTGGACTACC<br>AGGGTATCTAATCCTGTTTGCTCCCCACGCTTTCGCGCCTCAGTGTCAGT<br>TACAGACCAGAAAGTCGCCTTCGCCACTGGTGTTCCTCCATATCTCTACG<br>CATTTCACCGCTACACATGGAATTCCACTTTCCTCTTCTGCACTCAAGTC<br>TCCCAGTTTCCAATGACCCTCCACGGTTGAGCCGTGGGCTTTCACATCAG<br>ACTTAAGAAACCACCTGCGCGCGCTTTACGCCCAATAATTCCGGATAACG<br>CTTGCCACCTACGTATTACCGCGGCTGCTGGCACGTAGTTAGCCGTGGCT<br>TTCTGGTTAGGTACCGTCAAGGTGCCAGCTTATTCAACTAGCACTTGTTC<br>TTCCCTAACAACAGAGTTTTACGACCCGAAAGCCTTCATCACTCACGCGG<br>CGTTGCTCCGTCAGACTTTCGTCCATTGCGGAAGATTCCCTACTGCTGCC<br>TCCCGTAGGAGTCTGGGCCGTGTCTCAGTCCCAGTGTGGCCGATCACCCT<br>CTCAGGTCGGCTACGCATCGTTGCCTTGGTGAGCCGTTACCTCACCAACT<br>AGCTAATGCGACGCGGGTCCATCCATAAGTGACAGCCGAAGCCGCCTTTC<br>AATTTCGAACCATGCAGTTCAAAATGTTATCCGGTATTAGCCCCGGTTTC<br>CCGGAGTTATCCCAGTCTTATGGGCAGGTTACCCACGTGTTACTCACCCG<br>TCCGCCGCTAACTTCATAAGAGCAAGCTCTTAATCCATTCGCTCGACTGC
**Honey_2**	AAAAAGGTTACCCCACCGACTTCGGGTGTTACAAACTCTCGTGGTGTGAC<br>GGGCGGTGTGTACAAGGCCCGGGAACGTATTCACCGCGGCATGCTGATCC<br>GCGATTACTAGCGATTCCAGCTTCATGTAGGCGAGTTGCAGCCTACAATC

	CGAACTGAGAACGGTTTTATGAGATTAGCTCCACCTCGCGGTCTTGCAGC<br>TCTTTGTACCGTCCATTGTAGCACGTGTGTAGCCCAGGTCATAAGGGGCA<br>TGATGATTTGACGTCATCCCCACCTTCCTCCGGTTTGTCACCGGCAGTCA<br>CCTTAGAGTGCCCAACTTAATGATGGCAACTAAGATCAAGGGTTGCGCTC<br>GTTGCGGGACTTAACCCAACATCTCACGACACGAGCTGACGACAACCATG<br>CACCACCTGTCACTCTGCTCCCGAAGGAGAAGCCCTATCTCTAGGGTTTT<br>CAGAGGATGTCAAGACCTGGTAAGGTTCTTCGCGTTGCTTCGAATTAAAC<br>CACATGCTCCACCGCTTGTGCGGGCCCCCGTCAATTCCTTTGAGTTTCAG<br>CCTTGCGGCCGTACTCCCCAGGCGGAGTGCTTAATGCGTTAACTTCAGCA<br>CTAAAGGGCGGAAACCCTCTAACACTTAGCACTCATCGTTTACGGCGTGG<br>ACTACCAGGGTATCTAATCCTGTTTGCTCCCCACGCTTTCGCGCCTCAGT<br>GTCAGTTACAGACCAGAAAGTCGCCTTCGCCACTGGTGTTCCTCCATATC<br>TCTACGCATTTCACCGCTACACATGGAATTCCACTTTCCTCTTCTGCACT<br>CAAGTCTCCCAGTTTCCAATGACCCTCCACGGTTGAGCCGTGGGCTTTCA<br>CATCAGACTTAAGAAACCACCTGCGCGCGCTTTACGCCCAATAATTCCGG<br>ATAACGCTTGCCACCTACGTATTACCGCGGCTGCTGGCACGTAGTTAGCC<br>GTGGCTTTCTGGTTAGGTACCGTCAAGGTGCCAGCTTATTCAACTAGCAC<br>TTGTTCTTCCCTAACAACAGAGTTTTACGACCCGAAAGCCTTCATCACTC<br>ACGCGGCGTTGCTCCGTCAGACTTTCGTCCATTGCGGAAGATTCCCTACT<br>GCTGCCTCCCGTAGGAGTCTGGGCCGTGTCTCAGTCCCAGTGTGGCCGAT<br>CACCCTCTCAGGTCGGCTACGCATCGTTGCCTTGGTGAGCCGTTACCTCA<br>CCAACTAGCTAATGCGACGCGGGTCCATCCATAAGTGACAGCCGAAGCCG<br>CCTTTCAATTTCGAACCATGCAGTTCAAAATGTTATCCGGTATTAGCCCC<br>GGTTTCCCGGAGTTATCCCAGTCTTATGGGCAGGTTACCCACGTGTTACT<br>CACCCGTCCGCCGCTAACTTCATAAGAGCAAGCTCTTAATCCATTCGCTC<br>GACTGG
**Pollen_1**	GCTGGCTCCAAAAGGTTACCCCACCGACTTCGGGTGTTACAAACTCTCGT<br>GGTGTGACGGGCGGTGTGTACAAGGCCCGGGAACGTATTCACCGCGGCAT<br>GCTGATCCGCGATTACTAGCGATTCCAGCTTCATGTAGGCGAGTTGCAGC<br>CTACAATCCGAACTGAGAACGGTTTTATGAGATTAGCTCCACCTCGCGGT<br>CTTGCAGCTCTTTGTACCGTCCATTGTAGCACGTGTGTAGCCCAGGTCAT<br>AAGGGGCATGATGATTTGACGTCATCCCCACCTTCCTCCGGTTTGTCACC<br>GGCAGTCACCTTAGAGTGCCCAACTTAATGATGGCAACTAAGATCAAGGG<br>TTGCGCTCGTTGCGGGACTTAACCCAACATCTCACGACACGAGCTGACGA<br>CAACCATGCACCACCTGTCACTCTGCTCCCGAAGGAGAAGCCCTATCTCT<br>AGGGTTTTCAGAGGATGTCAAGACCTGGTAAGGTTCTTCGCGTTGCTTCG<br>AATTAAACCACATGCTCCACCGCTTGTGCGGGCCCCCGTCAATTCCTTTG<br>AGTTTCAGCCTTGCGGCCGTACTCCCCAGGCGGAGTGCTTAATGCGTTAA<br>CTTCAGCACTAAAGGGCGGAAACCCTCTAACACTTAGCACTCATCGTTTA<br>CGGCGTGGACTACCAGGGTATCTAATCCTGTTTGCTCCCCACGCTTTCGC<br>GCCTCAGTGTCAGTTACAGACCAGAAAGTCGCCTTCGCCACTGGTGTTCC<br>TCCATATCTCTACGCATTTCACCGCTACACATGGAATTCCACTTTCCTCT<br>TCTGCACTCAAGTCTCCCAGTTTCCAATGACCCTCCACGGTTGAGCCGTG<br>GGCTTTCACATCAGACTTAAGAAACCACCTGCGCGCGCTTTACGCCCAAT<br>AATTCCGGATAACGCTTGCCACCTACGTATTACCGCGGCTGCTGGCACGT<br>AGTTAGCCGTGGCTTTCTGGTTAGGTACCGTCAAGGTGCCAGCTTATTCA<br>ACTAGCACTTGTTCTTCCCTAACAACAGAGTTTTACGACCCGAAAGCCTT<br>CATCACTCACGCGGCGTTGCTCCGTCAGACTTTCGTCCATTGCGGAAGAT<br>TCCCTACTGCTGCCTCCCGTAGGAGTCTGGGCCGTGTCTCAGTCCCAGTG<br>TGGCCGATCACCCTCTCAGGTCGGCTACGCATCGTTGCCTTGGTGAGCCG<br>TTACCTCACCAACTAGCTAATGCGACGCGGGTCCATCCATAAGTGACAGC<br>CGAAGCCGCCTTTCAATTTCGAACCATGCAGTTCAAAATGTTATCCGGTA<br>TTAGCCCCGGTTTCCCGGAGTTATCCCAGTCTTATGGGCAGGTTACCCAC<br>GTGTTACTCACCCGTCCGCCGCTAACTTCATAAGAGCAAGCTCTTAATCC<br>ATTCGCTCGACTTGCATG
**Pollen_2**	GGCTGGCTCCAAAAGGTTACCCCACCGACTTCGGGTGTTACAAACTCTCG<br>TGGTGTGACGGGCGGTGTGTACAAGGCCCGGGAACGTATTCACCGCGGCA<br>TGCTGATCCGCGATTACTAGCGATTCCAGCTTCATGTAGGCGAGTTGCAG<br>CCTACAATCCGAACTGAGAACGGTTTTATGAGATTAGCTCCACCTCGCGG<br>TCTTGCAGCTCTTTGTACCGTCCATTGTAGCACGTGTGTAGCCCAGGTCA<br>TAAGGGGCATGATGATTTGACGTCATCCCCACCTTCCTCCGGTTTGTCAC<br>CGGCAGTCACCTTAGAGTGCCCAACTTAATGATGGCAACTAAGATCAAGG<br>GTTGCGCTCGTTGCGGGACTTAACCCAACATCTCACGACACGAGCTGACG<br>ACAACCATGCACCACCTGTCACTCTGCTCCCGAAGGAGAAGCCCTATCTC<br>TAGGGTTTTCAGAGGATGTCAAGACCTGGTAAGGTTCTTCGCGTTGCTTC<br>GAATTAAACCACATGCTCCACCGCTTGTGCGGGCCCCCGTCAATTCCTTT<br>GAGTTTCAGCCTTGCGGCCGTACTCCCCAGGCGGAGTGCTTAATGCGTTA

<table>
<tr><td></td><td>ACTTCAGCACTAAAGGGCGGAAACCCTCTAACACTTAGCACTCATCGTTT
ACGGCGTGGACTACCAGGGTATCTAATCCTGTTTGCTCCCCACGCTTTCG
CGCCTCAGTGTCAGTTACAGACCAGAAAGTCGCCTTCGCCACTGGTGTTC
CTCCATATCTCTACGCATTTCACCGCTACACATGGAATTCCACTTTCCTC
TTCTGCACTCAAGTCTCCCAGTTTCCAATGACCCTCCACGGTTGAGCCGT
GGGCTTTCACATCAGACTTAAGAAACCACCTGCGCGCGCTTTACGCCCAA
TAATTCCGGATAACGCTTGCCACCTACGTATTACCGCGGCTGCTGGCACG
TAGTTAGCCGTGGCTTTCTGGTTAGGTACCGTCAAGGTGCCAGCTTATTC
AACTAGCACTTGTTCTTCCCTAACAACAGAGTTTTACGACCCGAAAGCCT
TCATCACTCACGCGGCGTTGCTCCGTCAGACTTTCGTCCATTGCGGAAGA
TTCCCTACTGCTGCCTCCCGTAGGAGTCTGGGCCGTGTCTCAGTCCCAGT
GTGGCCGATCACCCTCTCAGGTCGGCTACGCATCGTTGCCTTGGTGAGCC
GTTACCTCACCAACTAGCTAATGCGACGCGGGTCCATCCATAAGTGACAG
CCGAAGCCGCCTTTCAATTTCGAACCATGCAGTTCAAAATGTTATCCGGT
ATTAGCCCCGGTTTCCCGGAGTTATCCCAGTCTTATGGGCAGGTTACCCA
CGTGTTACTCACCCGTCCGCCGCTAACTTCATAAGAGCAAGCTCTTAATC
CATTCGCTCGACTGCATGTA</td></tr>
<tr><td>Pollen_3</td><td>GCTGGCTCCAAAAGGTTACCCCACCGACTTCGGGTGTTACAAACTCTCGT
GGTGTGACGGGCGGTGTGTACAAGGCCCGGGAACGTATTCACCGCGGCAT
GCTGATCCGCGATTACTAGCGATTCCAGCTTCATGTAGGCGAGTTGCAGC
CTACAATCCGAACTGAGAACGGTTTTATGAGATTAGCTCCACCTCGCGGT
CTTGCAGCTCTTTGTACCGTCCATTGTAGCACGTGTGTAGCCCAGGTCAT
AAGGGGCATGATGATTTGACGTCATCCCCACCTTCCTCCGGTTTGTCACC
GGCAGTCACCTTAGAGTGCCCAACTTAATGATGGCAACTAAGATCAAGGG
TTGCGCTCGTTGCGGGACTTAACCCAACATCTCACGACACGAGCTGACGA
CAACCATGCACCACCTGTCACTCTGCTCCCGAAGGAGAAGCCCTATCTCT
AGGGTTTTCAGAGGATGTCAAGACCTGGTAAGGTTCTTCGCGTTGCTTCG
AATTAAACCACATGCTCCACCGCTTGTGCGGGCCCCCGTCAATTCCTTTG
AGTTTCAGCCTTGCGGCCGTACTCCCCAGGCGGAGTGCTTAATGCGTTAA
CTTCAGCACTAAAGGGCGGAAACCCTCTAACACTTAGCACTCATCGTTTA
CGGCGTGGACTACCAGGGTATCTAATCCTGTTTGCTCCCCACGCTTTCGC
GCCTCAGTGTCAGTTACAGACCAGAAAGTCGCCTTCGCCACTGGTGTTCC
TCCATATCTCTACGCATTTCACCGCTACACATGGAATTCCACTTTCCTCT
TCTGCACTCAAGTCTCCCAGTTTCCAATGACCCTCCACGGTTGAGCCGTG
GGCTTTCACATCAGACTTAAGAAACCACCTGCGCGCGCTTTACGCCCAAT
AATTCCGGATAACGCTTGCCACCTACGTATTACCGCGGCTGCTGGCACGT
AGTTAGCCGTGGCTTTCTGGTTAGGTACCGTCAAGGTGCCAGCTTATTCA
ACTAGCACTTGTTCTTCCCTAACAACAGAGTTTTACGACCCGAAAGCCTT
CATCACTCACGCGGCGTTGCTCCGTCAGACTTTCGTCCATTGCGGAAGAT
TCCCTACTGCTGCCTCCCGTAGGAGTCTGGGCCGTGTCTCAGTCCCAGTG
TGGCCGATCACCCTCTCAGGTCGGCTACGCATCGTTGCCTTGGTGAGCCG
TTACCTCACCAACTAGCTAATGCGACGCGGGTCCATCCATAAGTGACAGC
CGAAGCCGCCTTTCAATTTCGAACCATGCAGTTCAAAATGTTATCCGGTA
TTAGCCCCGGTTTCCCGGAGTTATCCCAGTCTTATGGGCAGGTTACCCAC
GTGTTACTCACCCGTCCGCCGCTAACTTCATAAGAGCAAGCTCTTAATCC
ATTCGCTCGACTGCATG</td></tr>
<tr><td>Comb_1</td><td>TGGCTCCAAAAAGGTTACCCCACCGACTTCGGGTGTTACAAACTCTCGTG
GTGTGACGGGCGGTGTGTACAAGGCCCGGGAACGTATTCACCGCGGCATG
CTGATCCGCGATTACTAGCGATTCCAGCTTCATGTAGGCGAGTTGCAGCC
TACAATCCGAACTGAGAACGGTTTTATGAGATTAGCTCCACCTCGCGGTC
TTGCAGCTCTTTGTACCGTCCATTGTAGCACGTGTGTAGCCCAGGTCATA
AGGGGCATGATGATTTGACGTCATCCCCACCTTCCTCCGGTTTGTCACCG
GCAGTCACCTTAGAGTGCCCAACTTAATGATGGCAACTAAGATCAAGGGT
TGCGCTCGTTGCGGGACTTAACCCAACATCTCACGACACGAGCTGACGAC
AACCATGCACCACCTGTCACTCTGCTCCCGAAGGAGAAGCCCTATCTCTA
GGGTTTTCAGAGGATGTCAAGACCTGGTAAGGTTCTTCGCGTTGCTTCGA
ATTAAACCACATGCTCCACCGCTTGTGCGGGCCCCCGTCAATTCCTTTGA
GTTTCAGCCTTGCGGCCGTACTCCCCAGGCGGAGTGCTTAATGCGTTAAC
TTCAGCACTAAAGGGCGGAAACCCTCTAACACTTAGCACTCATCGTTTAC
GGCGTGGACTACCAGGGTATCTAATCCTGTTTGCTCCCCACGCTTTCGCG
CCTCAGTGTCAGTTACAGACCAGAAAGTCGCCTTCGCCACTGGTGTTCCT
CCATATCTCTACGCATTTCACCGCTACACATGGAATTCCACTTTCCTCTT
CTGCACTCAAGTCTCCCAGTTTCCAATGACCCTCCACGGTTGAGCCGTGG
GCTTTCACATCAGACTTAAGAAACCACCTGCGCGCGCTTTACGCCCAATA
ATTCCGGATAACGCTTGCCACCTACGTATTACCGCGGCTGCTGGCACGTA
GTTAGCCGTGGCTTTCTGGTTAGGTACCGTCAAGGTGCCAGCTTATTCAA
CTAGCACTTGTTCTTCCCTAACAACAGAGTTTTACGACCCGAAAGCCTTC</td></tr>
</table>

	ATCACTCACGCGGCGTTGCTCCGTCAGACTTTCGTCCATTGCGGAAGATT<br>CCCTACTGCTGCCTCCCGTAGGAGTCTGGGCCGTGTCTCAGTCCCAGTGT<br>GGCCGATCACCCTCTCAGGTCGGCTACGCATCGTTGCCTTGGTGAGCCGT<br>TACCTCACCAACTAGCTAATGCGACGCGGGTCCATCCATAAGTGACAGCC<br>GAAGCCGCCTTTCAATTTCGAACCATGCAGTTCAAAATGTTATCCGGTAT<br>TAGCCCCGGTTTCCCGGAGTTATCCCAGTCTTATGGGCAGGTTACCCACG<br>TGTTACTCACCCGTCCGCCGCTAACTTCATAAGAGCAAGCTCTTAATCC
**Comb_2**	TGGCTCCAAAAAGGTTACCCCACCGACTTCGGGTGTTACAAACTCTCGTG<br>GTGTGACGGGCGGTGTGTACAAGGCCCGGGAACGTATTCACCGCGGCATG<br>CTGATCCGCGATTACTAGCGATTCCAGCTTCATGTAGGCGAGTTGCAGCC<br>TACAATCCGAACTGAGAACGGTTTTATGAGATTAGCTCCACCTCGCGGTC<br>TTGCAGCTCTTTGTACCGTCCATTGTAGCACGTGTGTAGCCCAGGTCATA<br>AGGGGCATGATGATTTGACGTCATCCCCACCTTCCTCCGGTTTGTCACCG<br>GCAGTCACCTTAGAGTGCCCAACTTAATGATGGCAACTAAGATCAAGGGT<br>TGCGCTCGTTGCGGGACTTAACCCAACATCTCACGACACGAGCTGACGAC<br>AACCATGCACCACCTGTCACTCTGCTCCCGAAGGAGAAGCCCTATCTCTA<br>GGGTTTTCAGAGGATGTCAAGACCTGGTAAGGTTCTTCGCGTTGCTTCGA<br>ATTAAACCACATGCTCCACCGCTTGTGCGGGCCCCCGTCAATTCCTTTGA<br>GTTTCAGCCTTGCGGCCGTACTCCCCAGGCGGAGTGCTTAATGCGTTAAC<br>TTCAGCACTAAAGGGCGGAAACCCTCTAACACTTAGCACTCATCGTTTAC<br>GGCGTGGACTACCAGGGTATCTAATCCTGTTTGCTCCCCACGCTTTCGCG<br>CCTCAGTGTCAGTTACAGACCAGAAAGTCGCCTTCGCCACTGGTGTTCCT<br>CCATATCTCTACGCATTTCACCGCTACACATGGAATTCCACTTTCCTCTT<br>CTGCACTCAAGTCTCCCAGTTTCCAATGACCCTCCACGGTTGAGCCGTGG<br>GCTTTCACATCAGACTTAAGAAACCACCTGCGCGCGCTTTACGCCCAATA<br>ATTCCGGATAACGCTTGCCACCTACGTATTACCGCGGCTGCTGGCACGTA<br>GTTAGCCGTGGCTTTCTGGTTAGGTACCGTCAAGGTGCCAGCTTATTCAA<br>CTAGCACTTGTTCTTCCCTAACAACAGAGTTTTACGACCCGAAAGCCTTC<br>ATCACTCACGCGGCGTTGCTCCGTCAGACTTTCGTCCATTGCGGAAGATT<br>CCCTACTGCTGCCTCCCGTAGGAGTCTGGGCCGTGTCTCAGTCCCAGTGT<br>GGCCGATCACCCTCTCAGGTCGGCTACGCATCGTTGCCTTGGTGAGCCGT<br>TACCTCACCAACTAGCTAATGCGACGCGGGTCCATCCATAAGTGACAGCC<br>GAAGCCGCCTTTCAATTTCGAACCATGCAGTTCAAAATGTTATCCGGTAT<br>TAGCCCCGGTTTCCCGGAGTTATCCCAGTCTTATGGGCAGGTTACCCACG<br>TGTTACTCACCCGTCCGCCGCTAACTTCATAAGAGCAAGCTCTTAATCCA<br>T
**Comb_3**	TGGCTCCAAAAAGGTTACCCCACCGACTTCGGGTGTTACAAACTCTCGTG<br>GTGTGACGGGCGGTGTGTACAAGGCCCGGGAACGTATTCACCGCGGCATG<br>CTGATCCGCGATTACTAGCGATTCCAGCTTCATGTAGGCGAGTTGCAGCC<br>TACAATCCGAACTGAGAACGGTTTTATGAGATTAGCTCCACCTCGCGGTC<br>TTGCAGCTCTTTGTACCGTCCATTGTAGCACGTGTGTAGCCCAGGTCATA<br>AGGGGCATGATGATTTGACGTCATCCCCACCTTCCTCCGGTTTGTCACCG<br>GCAGTCACCTTAGAGTGCCCAACTTAATGATGGCAACTAAGATCAAGGGT<br>TGCGCTCGTTGCGGGACTTAACCCAACATCTCACGACACGAGCTGACGAC<br>AACCATGCACCACCTGTCACTCTGCTCCCGAAGGAGAAGCCCTATCTCTA<br>GGGTTTTCAGAGGATGTCAAGACCTGGTAAGGTTCTTCGCGTTGCTTCGA<br>ATTAAACCACATGCTCCACCGCTTGTGCGGGCCCCCGTCAATTCCTTTGA<br>GTTTCAGCCTTGCGGCCGTACTCCCCAGGCGGAGTGCTTAATGCGTTAAC<br>TTCAGCACTAAAGGGCGGAAACCCTCTAACACTTAGCACTCATCGTTTAC<br>GGCGTGGACTACCAGGGTATCTAATCCTGTTTGCTCCCCACGCTTTCGCG<br>CCTCAGTGTCAGTTACAGACCAGAAAGTCGCCTTCGCCACTGGTGTTCCT<br>CCATATCTCTACGCATTTCACCGCTACACATGGAATTCCACTTTCCTCTT<br>CTGCACTCAAGTCTCCCAGTTTCCAATGACCCTCCACGGTTGAGCCGTGG<br>GCTTTCACATCAGACTTAAGAAACCACCTGCGCGCGCTTTACGCCCAATA<br>ATTCCGGATAACGCTTGCCACCTACGTATTACCGCGGCTGCTGGCACGTA<br>GTTAGCCGTGGCTTTCTGGTTAGGTACCGTCAAGGTGCCAGCTTATTCAA<br>CTAGCACTTGTTCTTCCCTAACAACAGAGTTTTACGACCCGAAAGCCTTC<br>ATCACTCACGCGGCGTTGCTCCGTCAGACTTTCGTCCATTGCGGAAGATT<br>CCCTACTGCTGCCTCCCGTAGGAGTCTGGGCCGTGTCTCAGTCCCAGTGT<br>GGCCGATCACCCTCTCAGGTCGGCTACGCATCGTTGCCTTGGTGAGCCGT<br>TACCTCACCAACTAGCTAATGCGACGCGGGTCCATCCATAAGTGACAGCC<br>GAAGCCGCCTTTCAATTTCGAACCATGCAGTTCAAAATGTTATCCGGTAT<br>TAGCCCCGGTTTCCCGGAGTTATCCCAGTCTTATGGGCAGGTTACCCACG<br>TGTTACTCACCCGTCCGCCGCTAACTTCATAAGAGCAAGCTCTTAATCCA<br>TTCGCTCGACTGCAT
